# Supplementary material for: Prevalence and correlates of pre-diabetes in adults of mixed ethnicities in the South African population: A systematic review and meta-analysis
Source: PLoS One. 2022 Nov 29;17(11):e0278347. doi: 10.1371/journal.pone.0278347 (PMC9707763; doi:10.1371/journal.pone.0278347)
Supplement: S2 Table — (DOCX) [file pone.0278347.s002.docx]

# **S2 Supplementary file**

| Table 1: Search strategy (PubMed) | |
| --- | --- |
| Search | **Search terms** |
| 4 | Search (#3 NOT animal[mh]) AND (“2000/01/01”[Date-Publication] : “2021/09/7”[Date-Publication]) |
| 3 | Search (#1 AND #2 AND #3) |
| 2 | Search (South Africa[mh] OR “South Africa*”[tiab] OR RSA[tiab] OR Southern Africa[tiab]) |
| 1 | Search (Diabetes OR Type 2 diabetes mellitus OR Hemoglobin A, glycosylated OR Pre-diabetes OR Glycosylated haemoglobin OR Impaired glucose tolerance OR Impaired fasting glucose |
