# Supplementary material for: Prevalence and correlates of pre-diabetes in adults of mixed ethnicities in the South African population: A systematic review and meta-analysis
Source: PLoS One. 2022 Nov 29;17(11):e0278347. doi: 10.1371/journal.pone.0278347 (PMC9707763; doi:10.1371/journal.pone.0278347)
Supplement: Attachment — Submitted filename: PLOS ONE SR PAPER REBUTTAL LETTER.docx [file pone.0278347.s003.docx]

10 September 2022

Dear Sir/Madam

**Re:** Prevalence and correlates of pre-diabetes in adults of mixed ethnicities in the South African population: A Systematic Review and Meta-analysis

Below is a table responding to the relevant comments from the editor and reviewer. The changes are found in the manuscript and highlighted in green for the editor and yellow for the reviewer.

# **Response to the editor table:**

| 1. Please ensure that your manuscript meets PLOS ONE's style requirements, including those for file naming. The PLOS ONE style templates can be found at | PLOS ONE style requirements have been met. |
| --- | --- |
| 2. Thank you for stating the following in the Acknowledgments Section of your manuscript:     "I would like to acknowledge the National Research Foundation for funding the study (Grant number:121558) and everyone in the Endorince research group for their academic and technical support." | The funding statement has been removed from the acknowledgements section. |
| 3. We note you have included a table to which you do not refer in the text of your manuscript. Please ensure that you refer to Table 2 in your text; if accepted, production will need this reference to link the reader to the Table. | Table 2 is referenced in the text. |
| 4. Please include captions for your Supporting Information files at the end of your manuscript, and update any in-text citations to match accordingly. Please see our Supporting Information guidelines for more information: | The supporting information heading was added at the end, and the in-text citations matched. |
|  |  |

# **Response to the reviewer table**

| Comments | Corrections |
| --- | --- |
| • More details are required to explain the association between key points and the conclusion. | More details were added in the conclusion in Page 23 (lines 2-3) & 23 (lines 10-13) |
| • Comprehensively explain the disease pathogenesis, its associated complications and the therapeutic interventions to strengthen your study e.g., https://doi.org/10.1002/jcb.26174 , https://doi.org/10.1111/jfbc.14228 and https://doi.org/10.1615/CritRevEukaryotGeneExpr.2015013358 | A comprehensive explanation has been added to the introduction. Lines 6 -24 on page 4 & lines 1-2 on page 5. |
| • Results are not well explained and comprehensively described. | The description of the results has been improved comprehensively. Lines 2-6 on page 17; page 14; lines 1-3 on page 16 |
| • More graphical representations of the results should be included in the manuscript. | Converted table 1 to a bar chart. |
| • More description is required in the discussion section to elaborate on the association between the results and the literature review. | More description was added on pages 19 (line 4 – 15) & 20 (line 8 – 13) |
| • Many references are missing or inconsistent in format. | References have been corrected. |
| • The format of the manuscript is inconsistent and is required per the requirement of the journal. | Corrected. |
| • There are some grammatical mistakes in the manuscript, such as verbs and prepositions. The manuscript needs extensive review by an author. | The paper has been reviewed thoroughly, and all grammatical errors have been corrected. |
